# Is Gut Microbiota Dysbiosis a Predictor of Increased Susceptibility to Poor Outcome of COVID-19 Patients? An Update

**DOI:** 10.3390/microorganisms9010053

**Published:** 2020-12-28

**Authors:** Carolina Ferreira, Sofia D. Viana, Flávio Reis

**Affiliations:** 1 Institute of Pharmacology & Experimental Therapeutics, & Coimbra Institute for Clinical and Biomedical Research (iCBR), Faculty of Medicine, University of Coimbra, 3000-548 Coimbra, Portugal; f202carolina@gmail.com (C.F.); sofia_viana@estescoimbra.pt (S.D.V.); 2 Center for Innovative Biomedicine and Biotechnology (CIBB), University of Coimbra, 3004-504 Coimbra, Portugal; 3 Clinical Academic Center of Coimbra (CACC), 3000-075 Coimbra, Portugal; 4 Polytechnic Institute of Coimbra, ESTESC-Coimbra Health School, Pharmacy, 3046-854 Coimbra, Portugal

**Keywords:** COVID-19, susceptibility to progress, gut microbiota dysbiosis, immune response, inflammation

## Abstract

The scientific knowledge already attained regarding the way severe acute respiratory syndrome coronavirus 2 (SARS-CoV-2) infects human cells and the clinical manifestations and consequences for Coronavirus Disease 2019 (COVID-19) patients, especially the most severe cases, brought gut microbiota into the discussion. It has been suggested that intestinal microflora composition plays a role in this disease because of the following: (i) its relevance to an efficient immune system response; (ii) the fact that 5–10% of the patients present gastrointestinal symptoms; and (iii) because it is modulated by intestinal angiotensin-converting enzyme 2 (ACE2) (which is the virus receptor). In addition, it is known that the most severely affected patients (those who stay longer in hospital, who require intensive care, and who eventually die) are older people with pre-existing cardiovascular, metabolic, renal, and pulmonary diseases, the same people in which the prevalence of gut microflora dysbiosis is higher. The COVID-19 patients presenting poor outcomes are also those in which the immune system’s hyperresponsiveness and a severe inflammatory condition (collectively referred as “cytokine storm”) are particularly evident, and have been associated with impaired microbiota phenotype. In this article, we present the evidence existing thus far that may suggest an association between intestinal microbiota composition and the susceptibility of some patients to progress to severe stages of the disease.

## 1. The “Gut Microbiota Hypothesis” in Poor Outcomes of COVID-19 Patients

Gut microbiota is a complex and dynamic ecosystem that comprises trillions of microorganisms, including bacteria and virus, with which the host maintains a beneficial symbiotic relationship [1,2,3]. This microbe community is extremely important in maintaining the host’s homeostasis, influencing several of its physiological functions, such as energy production, maintenance of the intestinal integrity, protection against pathogenic organisms, and regulation of host’s immunity [2,3,4,5,6]. However, these homeostasis mechanisms can become compromised as a consequence of alterations in the normal gut microbiota composition or functions, a condition known as dysbiosis [7]. Gut microbiota is influenced by different factors, both environmental and intrinsic to the host [3], including geographic localization, diet and nutrition, aging, antibiotics’ intake, stress, as well as by disease states, among other factors [3,6,8,9,10]. Changes in intestinal microbiota composition towards dysbiosis will affect and compromise the host’s functions in which it is involved, including immune system response against infections. On the other hand, there is evidence that infections, including bacterial or viral, can cause alterations in the intestinal flora, predisposing the host to secondary infections and aggravating its clinical status [2,11,12,13].

The year 2020 will be remembered in history for the emergence of millions of infections caused by a new virus from the Coronavirus family, named severe acute respiratory syndrome coronavirus 2 (SARS-CoV-2). This infection, designated by the World Health Organization (WHO) as Coronavirus Disease 2019 (COVID-19), has been disseminating all over the world, reaching pandemic proportions. In about a year, the infection has already affected more than 60 million people from almost all countries and caused more than 1.5 million deaths, as of December 2020.

SARS-CoV-2 infection starts by the binding of virus spike surface glycoprotein (S) to angiotensin-converting enzyme 2 (ACE2) receptors present in many human cells, which is then cleaved by host proteases (e.g., cathepsin, TMPRRS2, or furin), thus allowing virus internalization in the host cells [14]. The most typical symptoms, which usually appear in a few days after viral exposure, are fever, cough, fatigue, muscle or body aches, and shortness of breath, further evolving to pneumonia. In more severe cases, patients present respiratory, hepatic, gastrointestinal, and neurological complications, which require hospitalization and eventually progress to multi-organ dysfunction and death [15]. COVID-19 severity and mortality rate are considerably higher in elderly patients, particularly those with pre-existing comorbidities, including hypertension, diabetes, renal disease, or pulmonary conditions, among other chronic diseases [16,17,18].

Additionally, different studies have demonstrated that 5–10% of COVID-19 patients present digestive symptoms, such as abdominal pain, vomits, and diarrhea, as well as intestinal inflammation [19,20,21,22]. These data suggest that the gastrointestinal tract might be a location of viral activity and replication, which agrees with the high expression of ACE2 in the intestinal epithelium [23,24,25,26]. ACE2 is recognized as an important regulator of the renin-angiotensin system (RAS) by counteracting the negative actions mediated by Angiotensin II signaling via its type 1 receptor [27]. Thus, cleavage of ACE2 after SARS-CoV-2 infection might contribute to explaining the poor outcomes observed in COVID-19 patients with pre-existing comorbidities usually associated with RAS overactivity, such as respiratory, cardiac, and renal disorders, as well as diabetes [27].

ACE2 also exerts non-RAS-related roles linked with the transport of neutral amino acids across the gut epithelial cells, with a putative impact on gut homeostasis and microbiota composition [28]. In fact, ACE2 acts as a chaperone for membrane trafficking of the amino acid transporter B0AT1, which mediates the uptake of neutral amino acids, namely tryptophan (Trp), into intestinal cells. A link between ACE2-mediated amino acid transport and gut flora composition has been suggested, in such a way that impaired ACE2 expression or function are potentially promoters of gut microbiota dysbiosis [28,29]. These pieces of evidence are in line with the gastrointestinal symptoms that have been reported in a non-negligible percentage of people with SARS-CoV-2, suggesting an impact on the gastrointestinal-enteric system [30]. In fact, several reports point to alterations in gut microflora composition in COVID-19 patients, with their microbiota being characterized by a decreased bacterial diversity, enrichment in opportunistic pathogens, and loss of beneficial symbionts [31,32,33,34,35]. Thus, it has been suggested that ACE2 shedding promoted by SARS-CoV-2 infection might contribute to intestinal microflora dysbiosis, thus eventually helping to explain the poor outcomes in COVID-19 patients with pre-existing comorbidities [36]. The infected patients with a higher frequency of intensive care unit (ICU) admission (disease severity) and increased mortality rate are typically elderly people with pre-existing cardiovascular, metabolic, and renal disorders, including hypertension, heart failure, myocardial infarction, stroke, coronary artery disease, diabetes, and chronic kidney disease, among others—conditions that have been associated with gut microbiota alterations [8,37,38].

In line with the previous major coronavirus outbreaks in humans (namely SARS-CoV and Middle East Respiratory Syndrome Coronavirus (MERS-CoV)) [39], the more severe cases of SARS-CoV-2 infection have been associated with a hyperresponse of the immune system, featured by an exacerbated systemic inflammatory response and the massive release of cytokines, collectively described as a “cytokine storm” [40]. The resulting multi-organ failure fueled by a self-sustaining loop of ongoing age-related immunosenescence and inflammaging can additionally contribute to the poor outcomes in elderly patients with chronic comorbidities [15,41,42].

Among other relevant metabolic and structural protective functions, gut microbiota plays a major role in the host immune system education and ability to respond to insults, including to infections [1]. Disruption of gut microbiota influences the host’s immune response, worsening SARS-CoV-2-induced injury, owing to an excessive reactivity of the immune system and a strong inflammatory state [43,44,45]. In addition, different lines of evidence show that respiratory viral infections may originate alterations in the intestinal microbiome composition, which predispose patients to secondary infections and aggravate their clinical status [11,12,43,44,45]. We and others have recently proposed that the triad of gut microbiota dysbiosis, immune hyperresponse, and inflammation could eventually explain why some COVID-19 patients are more resilient, while others are more fragile when infected with SARS-CoV-2, recovering faster or progressing to more severe clinical condition, respectively [25,46,47,48,49]. As the ongoing studies reveal new evidence, this hypothesis has been gaining more and more consistency, in such a way that gut microbiota composition might eventually be viewed as a putative predictor of COVID-19 susceptibility and severity. In the next paragraphs, we report the data already known that may contribute to validating this possibility.

## 2. What Is the Evidence So Far that Links Gut Microbiota Composition to COVID-19 Severity?

Gut microbiota is crucial to the process of development and function of the immune system [1,50,51,52], as it modulates immune cells towards anti- or pro-inflammatory responses. Different studies have described significant changes regarding the innate and adaptive immune systems in COVID-19 patients [53,54,55,56]. The cytokine storm, in particular, clearly reflects an uncontrolled dysregulation of the host’s immune function. Several pieces of evidence point to the occurrence of lymphocytopenia in individuals with SARS-CoV-2 [57,58,59,60,61]. In a study involving 452 severe COVID-19 patients in Wuhan, a significant decrease in the number of T lymphocytes, including helper and suppressor T cells, was observed [62]. Particularly, among helper T cells, the researchers reported a decrease in regulatory and memory T cells counts. However, naïve T cells percentage was increased in COVID-19 patients relative to healthy individuals, which might contribute to hyperinflammation events, as there is an imbalance between the activity of naïve T cells and that of regulatory and memory T cells [62]. Furthermore, a reduced number of memory T cells might be implicated in COVID-19 relapses, which are particularly evident when recurrences in recovered patients arise [62].

Kalfaoglu et al., by analyzing CD4+ T lymphocytes’ transcriptomes from bronchoalveolar lavage fluid (BALF) belonging to moderate and severe COVID-19 patients, observed that SARS-CoV-2 is capable of inducing activation and differentiation processes in these cells, accelerating both their activation and death [55]. These authors proposed a hypothesis stating that the abnormally activated CD4+ T cells might be able to promote the viral entry through Furin production in critically ill patients. When compared with moderate patients, CD4+ T cells from severe patients present an increased expression of the genes *fos*, *fosb*, and *jun*; of the activation marker MKI67; of Th2-related genes *maf* and *il4r*; and of chemokines CCL2, CCL3, CCL4, CCL7, CCL8, and CXCL8. These results suggest that CD4+ T cells in severe COVID-19 patients’ lungs are highly activated and recruit other immune cells. In contrast, these patients display decreased expression of interferon-induced genes, such as *ifit1*, *ifit2, ifit3*, and *ifim1*, as well as genes associated with interferon downstream pathways, suggesting that interferons might be suppressed in severe COVID-19 cases [55]. In addition, Huang et al. discovered that the plasmatic concentrations of interleukin (IL)-1β, IL-1ra, IL-7, IL-8, IL-9, IL-10, basic FGF, GM-CSF, G-CSF, VEGF, IP-10, MCP-1, IFN-γ, IFN-α, MIP-1α, and MIP-1β were higher in COVID-19 patients present in ICU, as well as non-ICU patients, when compared with healthy individuals [63]. A possible explanation for the potential contradictory evidence of a decrease in the expression of interferon-induced genes’ and interferon downstream pathways and increased plasmatic IFNs levels might be associated with the fact that plasma levels are defined by the IFNs’ input from several tissues, including the gut [64]. This study raises the premise that the cytokine storm observed in COVID-19 cases might be correlated with disease severity, as IL-2, IL-7, IL-10, G-CSF, MCP-1, IP-10, TNF-α, and MIP-1α levels were higher in ICU patients compared with non-ICU ones [63]. Moreover, a study evaluating a cohort of 44 hospitalized COVID-19 patients reported the existence of higher median fecal levels of IL-8 and lower levels of fecal IL-10 in COVID-19 patients compared with control individuals [65]. Furthermore, IL-23 fecal levels were increased in severe patients, suggesting the involvement of the GI tract in the SARS-CoV-2 infection in an immunological manner [65]. More severe cases were also associated with higher serum levels of IL-6, IL-8, tumor necrosis factor α (TNF-α), C-reactive protein (CRP), lactate dehydrogenase (LDH), D-dimer, ferritin, and procalcitonin [65]. Furthermore, a study performed by Li et al. allowed to observe that the lower the counts on admission of total T cell, CD4+ T cell, and CD8+ T cell, the more serious the disease and the worse the prognosis of the patients [66]. A recent study also showed that lymphopenia and an increase in cytokine levels were significantly correlated with disease severity, with the IL-2R/lymphocyte ration being a potential biomarker for COVID-19 disease severity and progression identification [67].

Several studies have already demonstrated that, when compared with healthy individuals, COVID-19 patients present a significantly reduced bacterial diversity [31,68]; higher abundancy of opportunistic bacteria such as Streptococcus, Rothia, Veilonella, and Actinomyces [31,34,35]; and decreased levels of benefic symbionts, including Agathobacter, Fusicatenibacter, Roseburia, and Ruminococcaceae UCG-013 [31,35]. A study performed by Zuo et al. reported that most patients’ gut microbiota composition alterations persisted even after viral clearance, suggesting that the infection or/and hospitalization might be associated with a long-lasting adverse effect regarding the composition of intestinal microflora community [35], which might be potentially associated with recovery delays. Remarkably, the existence of a correlation between the COVID-19 severity grade and the basal fecal microbiome has been established [35,68]. In the study performed by Zuo et al., twenty-three bacterial taxa showed a significant positive correlation with disease severity; with the main bacteria presenting a positive association with COVID-19 severity belong to the filo Firmicutes and the genus Coprobacillus, as well as the Clostridium ramosum and Clostridium hathewayi species [35]. Interestingly, the fact that Firmicutes presented this positive association with disease severity is in accordance with evidence showing that these bacteria possess a specific role in regulating ACE2 expression in the murine gut [69]. On the other hand, two beneficial bacterial species—Alistipes ondedonkii (important for the maintenance of intestinal homeostais) and Faecalibacterium prausnitzii (anti-inflammatory properties detainer)—showed a negative correlation with COVID-19 severity [35].

It is now acknowledged that gut microbiota is responsible for regulating several hosts’ physiological functions [70,71,72]. Particularly, numerous studies have reported that intestinal microflora affects lung health through a bidirectional pathway designated as the “gut–lung axis” [73,74,75]. One of the main complications associated with COVID-19 is acute respiratory distress syndrome (ARDS) [76,77], in which microbiota composition and function might play an important part. An enrichment of lung microbiota with intestinal Bacteroides species is observed in many COVID-19 cases [78], an event linked to increased plasmatic inflammatory markers levels [78]. Another study reported an increase in Enterobacteiaceae and Lachnospiraceae levels in severely ill patients with ARDS, when compared with patients that did not present this condition [32,79]. These results suggest that the microbiota could be seen as potential marker to predict ARDS and other possible scenarios associated with COVID-19 pathology.

Therefore, gut microbiota might provide information about the individual susceptibility to COVID-19. Gou et al. recently reported that changes in the normal composition and function of intestinal microbiota might predispose healthy individuals to an atypical inflammatory response, such as the one observed in COVID-19 cases [80]. Additionally, a study performed in Wuhan, China confirmed the existence of this relationship between gut microflora composition and the predisposition of healthy individuals to SARS-CoV-2 infection [78]. The researchers observed that individuals that display increased numbers of Lactobacillus present higher levels of IL-10, an anti-inflammatory cytokine, and generally a more favorable prognosis. In contrast, individuals displaying higher numbers of pro-inflammatory bacterial species, such as Klebsiella, Streptococcus, and Ruminococcus gnavus, showed increased levels of pro-inflammatory cytokines as well as more pronounced disease severity [78]. Furthermore, through the evaluation of the metabolomic and proteomic profile of COVID-19 patients’ serum, a study performed by Shen et al. revealed specific alterations in severely ill patients [33]. In fact, increased serum concentrations of inflammatory markers, such as IL-1β, IL-6, TNF-α, and CRP, were associated with a higher prognostic risk score (PRS) in patients over 58 years old [33]. By investigating the potential role of gut microbiota on the susceptibility of healthy individuals to COVID-19, the investigators demonstrated that the observed alterations regarding blood proteomic markers would be preceded by intestinal microflora changes, suggesting a potential causal relationship in the case of older patients [33] Specifically, the genus Bacteroides and Streptococcus, as well as the order Clostridiales, showed a negative correlation with the majority of the tested cytokines (IL-1β, IL-2, IL-4, IL-6, IL-8, IL-10, IL-12p70, IL-13, TNF-α, and IFN-γ), while the genus Lactobacillus, Ruminococcus, and Blautia displayed positive associations with the referred cytokines [33]. Another study has reported that abundant bacteria in COVID-19 patients, including Rothia, Streptococcus, Veilonella, and Actinomyces, are positively correlated with high levels of CRP and D-dimer, once more evidencing the influence of gut microbiota composition in the host’s inflammatory profile [31]. However, no significant alterations were observed in gut microbiota composition between patients with different disease severity stages [65]. These results suggest that, in this complex scenario of interactions between different systems (namely intestinal microbiota–immune system–inflammatory response), there may be additional factors playing a relevant role for disease severity. On the other hand, it also suggests that other elements able to shape microbiota should be carefully considered, including age; comorbidities; and especially the impact of drugs, particularly antibiotics, as highlighted in the study of Britton et al. [65].

Collectively, there is evidence suggesting that microbiota characteristics and related metabolites should be more profoundly investigated as potential prediction markers of individual susceptibility of COVID-19 patients to develop a more severe phenotype. Table 1 summarizes the major findings regarding gut microbiota, inflammation, and immune system changes in COVID-19 patients, and the suggested associations with disease severity. However, only the publication of more results from the different clinical trials related to gut microbiota with patients affected with distinct levels of severity could open up the possibility to clarify the existence of causality in this association.

## 3. Concluding Remarks and Future Directions

**Figure 1:**
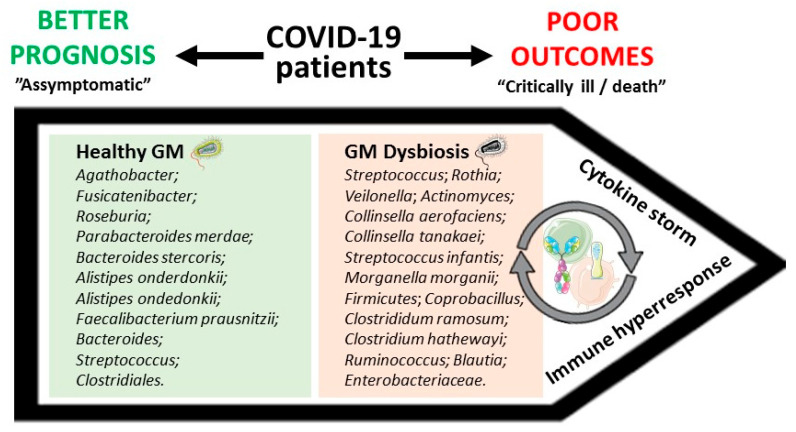
Putative correlation between Coronavirus Disease 2019 (COVID-19) clinical outcomes and gut microbiota (GM) composition. Green and red squares display some examples of bacteria encompassing better or poor COVID-19 outcomes, respectively.

COVID-19 patients display immune response deregulation and increased levels of specific inflammatory cytokines and chemokines, with these alterations being particularly intense in severe patients, in a condition often referred as cytokine storm [32,33,53,55]. Other studies also report that the blood lymphocyte percentage might reflect disease progression and severity [59], as well as the number of leukocytes and B and natural killer (NK) cells [81,82]. Gut microbiota plays major functions in the host, including immune system education and strengthening [1,51,52]. Several studies have reported major impairment of innate and adaptive immune systems in COVID-19 patients [53,54,55,56], accompanied by changes in gut microbiota composition [31,32,33,34,35]. It has been suggested that intestinal microflora composition could be correlated with the predisposition of healthy individuals to COVID-19 and with disease severity [31,33,34,35,78]. In particular, some data suggest that certain microbiota characteristics allow the prediction of the occurrence of ARDS and other disease-associated scenarios [32,78]. Moreover, COVID-19 patients’ microbial composition correlates with altered levels of inflammatory markers when compared with healthy individuals [31,33,78], reinforcing the potential relevance for the disease. These data have been leading researchers to refer to gut microbiota composition, inflammatory markers’ levels, and immune cells’ number and activity as potential predictors of susceptibility of healthy individuals to COVID-19, as well as of disease severity (Figure 1), as these parameters differ significantly between healthy and infected individuals, as well as between moderate and severe COVID-19 patients [31,32,33,35,59,78,80]. However, with the current knowledge, it is impossible to ensure a causal relationship, which remains an open hypothesis that deserves to be better dissected.

The evidence collected thus far suggests that modifications in the characteristics of the intestinal microbial community and the relationship it establishes with the immune system, which leads to changes in inflammatory markers’ levels and in the number and function of several immune cells, should be more profoundly investigated as potential predictors of individual susceptibility to a more severe COVID-19 phenotype. Additionally, these parameters might be used to support the implementation of therapeutic measures to prevent disease evolution in populations with higher susceptibility. Critically ill patients on mechanical ventilation who were given probiotics, specifically Lactobacillus rhamnosus GG, live Bacillus subtilis, and Enterococcus faecalis, presented improvement of pneumonia when compared with placebo, in two randomized controlled trials [83,84]. However, the efficacy of probiotics use in COVID-19 patients remains to be proved and the issue is under debate [85,86], deserving more attention by the scientific-medical community.

## Figures and Tables

**Table 1:** Major findings regarding changes on gut microbiota, inflammation, and immune system markers in Coronavirus Disease 2019 (COVID-19) patients, and the suggested associations with disease severity.

Study Sample	Major Findings Regarding Gut Microbiota, Inflammation and Immune System Changes	Ref.
30 COVID-19 patients, 24 H1N1 patients, and 30 matched controls.	(i) Compared with healthy controls, COVID-19 patients displayed a depletion of *Agathobacter, Fusicatenibacter, Roseburia,* and *Ruminocaccaceae UCG-013*, whose abundancy was negatively correlated, mainly, with the levels of CRP, PCT, and D-dimer; (ii) CRP and D-dimer levels were positively correlated with bacteria abundance in COVID-19 cases, including *Streptococcus, Rothia, Veilonella*, and *Actinomyces*.	[31]
15 hospitalized COVID-19 patients, of which 7 had stool positivity for SARS-CoV-2.	(i) Faecal samples with high SARS-CoV-2 infectivity had higher abundances of *Collinsella aerofaciens, Collinsella tanakaei, Streptococcus infantis,* and *Morganella morganii*, and higher functional capacity for nucleotide de novo biosynthesis, amino acid biosynthesis, and glycolysis; (ii) faecal samples with signature of low-to-none SARS-CoV-2 infectivity had higher abundances of SCFAs-producing bacteria, *Parabacteroides merdae, Bacteroides stercoris, Alistipes onderdonkii*, and *Lachnospiraceae bacterium 1_1_57FAA*.	[34]
15 COVID-19 patients, 6 patients with pneumonia, and 15 healthy controls.	(i) In a sample of seven antibiotic-naïve patients, 23 bacterial taxa were discovered to correlate with disease severity; (ii) the main bacteria positively correlated with disease severity belonged to the phylum *Firmicutes, Coprobacillus* genus, and *Clostrididum ramosum* and *Clostridium hathewayi* species; (iii) the main bacteria negatively correlated with disease severity were *Alistipes ondedonkii* and *Faecalibacterium prausnitzii.*	[35]
COVID-19 patients (moderate vs. severe).	(i) Versus moderate patients, CD4^+^ T-cells from severe COVID-19 patients expressed higher levels of the AP-1 genes *fos, fosb*, and *jun*; the activation marker MKI67 (Ki67); Th2-related genes *il4r* and *maf*; and chemokines including CCL2, CCL3, CCL4, CCL7, CCL8, and CXCL8; (ii) CD4+ T-cells in severe patients expressed higher levels of immunoregulatory genes including immune checkpoints (*ctla4, havcr2 [tim-3],* and *lgals3 [galectin-3]*) as well as the Tregs and T-cell activation marker IL2RA (CD25); (iii) CD4+ T-cells from severe patients showed decreased expression of interferon-induced genes (*ifit1, ifit2, ifit3*, and *ifitm1*) and genes related to downstream pathways.	[55]
12 COVID-19 death cases (moderate vs. severe).	(i) Among all parameters studied, blood lymphocyte percentage (LYM%) showed the most significant and consistent trend, suggesting it as an indicator of disease progression; (ii) LYM% of severe patients decreased initially and then increased to >10% until being discharged; (iii) LYM% of moderate patients fluctuated very little after disease onset and was >20% when discharged.	[59]
522 COVID-19 patients, 43 of which were admitted in the ICU; vs. 40 healthy controls.	(i) In the severe and critical disease patients, as well as the perished ones, total T cells, CD4^+^, and CD8^+^ T cells were significantly lower than in the mild/moderate patients; (ii) total T cells, CD8^+^ T, or CD4^+^ T cells lower than 800, 300, and 400/μL, respectively, were negatively correlated with patient survival; (iii) T cell numbers were negatively correlated with serum IL-6, IL-10, and TNF-α levels, which were significantly higher in ICU patients than in non-ICU ones; (iv) increasing PD-1 and Tim-3 expression on T cells in patients progressed to symptomatic stages.	[61]
39 COVID-19 patients.	(i) The more serious the disease and the worse the prognosis, the lower the T cell, CD4+ *T* cell, and CD8+ *T* cell counts on admission.	[66]
452 COVID-19 patients: 166 non-severe; 286 severe.	(i) With increased severity of illness, leukocytes, neutrophils, infection biomarkers (CRP, PCT, and ferritin) and cytokines (IL-2R, IL-6, IL-8, IL-10, and TNF-α) were significantly increased, while lymphocytes were significantly decreased; (ii) the ratio of IL-2R to lymphocytes was found to be remarkably increased in severe and critical patients. IL-2R/lymphocytes ratio was superior vs. other markers for the identification of critical illness COVID-19; (iii) cytokine profile and IL-2R/lymphocytes were significantly decreased in recovered patients.	[67]
91 critically ill patients, within 24 h of their ICU admission.	(i) ARDS patients display an increased number of *Lachnospiraceae* and *Enterobacteriaceae*, which predicted fewer ventilator-free days; (ii) enrichment of the lungs with gut-associated bacteria was positively correlated with high levels of inflammatory markers, particularly TNF-α; (iii) increased *Lachnospiraceae* was a strong predictor of reduced survival in ARDS patients.	[79]
31 COVID-19 patients (18 non-severe cases and 13 severe cases) and 990 non-COVID-19 individuals.	(i) Each 10% increment in PRS was associated with a 57% higher risk of progressing to severe phases, as indicated by proteomic biomarkers SAA1, SAA2, SAA4, SERPINA3, C6, and CFB; (ii) higher PRS significantly correlated with increased serum levels of all the studied inflammatory markers among older healthy individuals (>58 years); (iii) in the same individuals, *Bacteroides* genus, *Streptococcus* genus, and *Clostridiales* order were negatively correlated with cytokines (IL-1β, IL-2, IL-4, IL-6, IL-8, IL-10, IL-12p70, IL-13, TNF-α, and IFN-γ), whereas *Ruminococcus* genus, *Blautia* genus, and *Lactobacillus* genus showed positive associations.	[80]

COVID-19, Coronavirus Disease 2019; H1N1, Influenza A vírus; CRP, C-reactive protein; PCT, procalcitonin; SARS-CoV-2, severe acute respiratory syndrome coronavirus 2; AP-1, activator protein 1; IL4R, interleukin 4 receptor; CCL, CC chemokine ligands; CXCL, chemokine (C-X-C motif) ligand; CTLA4, cytotoxic T-tymphocyte associated protein 4; HAVCR2, hepatitis A virus cellular receptor 2; TIM-3, T-cell immunoglobulin and mucin-domain containing-3; Tregs, regulatory T cells; IL2R, interleukin 2 (IL2) receptor; IL2RA, interleukin 2 (IL2) receptor alpha; CD25, α chain of the high-affinity IL-2 receptor; IFTI, interferon induced protein with tetratricopeptide repeats; IFITM1, interferon induced transmembrane protein 1; IL, interleukin; TNF-α, tumor necrosis factor α; ICU, intensive care unit; PD-1, programmed death-1; PRS, prognostic risk score; SAA, serum amyloid A; SERPINA3, α-1-antichymotrypsin; C6, complement component 6; CFB, complement factor B; IFN-γ, interferon gamma; ARDS, acute respiratory distress syndrome.

## References

[1] Belkaid Y., Hand T.W. (2014). Role of the microbiota in immunity and inflammation. Cell.

[2] Li N., Ma W.T., Pang M., Fan Q.L., Hua J.L. (2019). The Commensal Microbiota and Viral Infection: A Comprehensive Review. Front. Immunol..

[3] Thursby E., Juge N. (2017). Introduction to the human gut microbiota. Biochem. J..

[4] Guinane C.M., Cotter P.D. (2013). Role of the gut microbiota in health and chronic gastrointestinal disease: Understanding a hidden metabolic organ. Ther. Adv. Gastroenterol..

[5] Singh A.K., Cabral C., Kumar R., Ganguly R., Rana H.K., Gupta A., Lauro M.R., Carbone C., Reis F., Pandey A.K. (2019). Beneficial Effects of Dietary Polyphenols on Gut Microbiota and Strategies to Improve Delivery Efficiency. Nutrients.

[6] Wang B., Yao M., Lv L., Ling Z., Li L. (2017). The Human Microbiota in Health and Disease. Engineering.

[7] Carding S., Verbeke K., Vipond D.T., Corfe B.M., Owen L.J. (2015). Dysbiosis of the gut microbiota in disease. Microb. Ecol. Health Dis..

[8] Fernandes R., Viana S.D., Nunes S., Reis F. (2019). Diabetic gut microbiota dysbiosis as an inflammaging and immunosenescence condition that fosters progression of retinopathy and nephropathy. Biochim. Biophys. Acta Mol. Basis Dis..

[9] Hasan N., Yang H. (2019). Factors affecting the composition of the gut microbiota, and its modulation. PeerJ.

[10] Moran-Ramos S., Lopez-Contreras B.E., Villarruel-Vazquez R., Ocampo-Medina E., Macias-Kauffer L., Martinez-Medina J.N., Villamil-Ramirez H., León-Mimila P., Del Rio-Navarro B.E., Ibarra-Gonzalez I. (2020). Environmental and intrinsic factors shaping gut microbiota composition and diversity and its relation to metabolic health in children and early adolescents: A population-based study. Gut Microbes.

[11] Groves H.T., Higham S.L., Moffatt M.F., Cox M.J., Tregoning J.S. (2020). Respiratory Viral Infection Alters the Gut Microbiota by Inducing Inappetence. mBio.

[12] Hanada S., Pirzadeh M., Carver K.Y., Deng J.C. (2018). Respiratory Viral Infection-Induced Microbiome Alterations and Secondary Bacterial Pneumonia. Front. Immunol..

[13] Yuan L., Hensley C., Mahsoub H.M., Ramesh A.K., Zhou P. (2020). Microbiota in viral infection and disease in humans and farm animals. Prog. Mol. Biol. Transl. Sci..

[14] Hoffmann M., Kleine-Weber H., Krüger N., Müller M., Drosten C., Pöhlmann S. (2020). The novel coronavirus 2019 (2019-nCoV) uses the SARS-coronavirus receptor ACE2 and the cellular protease TMPRSS2 for entry into target cells. bioRxiv.

[15] Wu T., Zuo Z., Kang S., Jiang L., Luo X., Xia Z., Liu J., Xiao X., Ye M., Deng M. (2020). Multi-organ Dysfunction in Patients with COVID-19: A Systematic Review and Meta-analysis. Aging Dis..

[16] COVID, Team CDC, and Response Team (2020). Severe Outcomes Among Patients with Coronavirus Disease 2019 (COVID-19)-United States, February 12-March 16, 2020. Morb. Mortal. Wkly. Rep..

[17] Roncon L., Zuin M., Rigatelli G., Zuliani G. (2020). Diabetic patients with COVID-19 infection are at higher risk of ICU admission and poor short-term outcome. J. Clin. Virol..

[18] Siordia J.A. (2020). Epidemiology and clinical features of COVID-19: A review of current literature. J. Clin. Virol..

[19] Du M., Cai G., Chen F., Christiani D.C., Zhang Z., Wang M. (2020). Multiomics Evaluation of Gastrointestinal and Other Clinical Characteristics of COVID-19. Gastroenterology.

[20] Jin X., Lian J.S., Hu J.H., Gao J., Zheng L., Zhang Y.M., Hao S.R., Jia H.Y., Cai H., Zhang X.L. (2020). Epidemiological, clinical and virological characteristics of 74 cases of coronavirus-infected disease 2019 (COVID-19) with gastrointestinal symptoms. Gut.

[21] Villapol S. (2020). Gastrointestinal symptoms associated with COVID-19: Impact on the gut microbiome. Transl. Res..

[22] Yang L., Tu L. (2020). Implications of gastrointestinal manifestations of COVID-19. Lancet Gastroenterol. Hepatol..

[23] Hanrahan T.P., Lubel J.S., Garg M. (2020). Lessons From COVID-19, ACE2, and Intestinal Inflammation: Could a Virus Trigger Chronic Intestinal Inflammation?. Clin. Gastroenterol. Hepatol..

[24] Li L.Y., Wu W., Chen S., Gu J.W., Li X.L., Song H.J., Du F., Wang G., Zhong C.Q., Wang X.Y. (2020). Digestive system involvement of novel coronavirus infection: Prevention and control infection from a gastroenterology perspective. J. Dig. Dis..

[25] Penninger J.M., Grant M.B., Sung J.J.Y. (2020). The Role of Angiotensin Converting Enzyme 2 in Modulating Gut Microbiota, Intestinal Inflammation, and Coronavirus Infection. Gastroenterology.

[26] Xu J., Chu M., Zhong F., Tan X., Tang G., Mai J., Lai N., Guan C., Liang Y., Liao G. (2020). Digestive symptoms of COVID-19 and expression of ACE2 in digestive tract organs. Cell Death Discov..

[27] Kuba K., Imai Y., Penninger J.M. (2013). Multiple functions of angiotensin-converting enzyme 2 and its relevance in cardiovascular diseases. Circ. J..

[28] Perlot T., Penninger J.M. (2013). ACE2—From the renin–angiotensin system to gut microbiota and malnutrition. Microbes Infect..

[29] Hashimoto T., Perlot T., Rehman A., Trichereau J., Ishiguro H., Paolino M., Sigl V., Hanada T., Hanada R., Lipinski S. (2012). ACE2 links amino acid malnutrition to microbial ecology and intestinal inflammation. Nature.

[30] Kotfis K., Skonieczna-Żydecka K. (2020). COVID-19: Gastrointestinal symptoms and potential sources of SARS-CoV-2 transmission. Anaesthesiol. Intensive Ther..

[31] Gu S., Chen Y., Wu Z., Chen Y., Gao H., Lv L., Guo F., Zhang X., Luo R., Huang C. (2020). Alterations of the Gut Microbiota in Patients with COVID-19 or H1N1 Influenza. Clin. Infect. Dis..

[32] He Y., Wang J., Li F., Shi Y. (2020). Main Clinical Features of COVID-19 and Potential Prognostic and Therapeutic Value of the Microbiota in SARS-CoV-2 Infections. Front. Microbiol..

[33] Shen B., Yi X., Sun Y., Bi X., Du J., Zhang C., Quan S., Zhang F., Sun R., Qian L. (2020). Proteomic and Metabolomic Characterization of COVID-19 Patient Sera. Cell.

[34] Zuo T., Liu Q., Zhang F., Lui G., Tso E., Yeoh Y.K., Chen Z., Boon S., Chan F.K.L., Chan P. (2020). Depicting SARS-CoV-2 faecal viral activity in association with gut microbiota composition in patients with COVID-19. Gut.

[35] Zuo T., Zhang F., Lui G.C.Y., Yeoh Y.K., Li A.Y.L., Zhan H., Wan Y., Chung A.C.K., Cheung C.P., Chen N. (2020). Alterations in Gut Microbiota of Patients With COVID-19 During Time of Hospitalization. Gastroenterology.

[36] Viana S.D., Nunes S., Reis F. (2020). ACE2 imbalance as a key player for the poor outcomes in COVID-19 patients with age-related comorbidities-Role of gut microbiota dysbiosis. Ageing Res. Rev..

[37] Busnelli M., Manzini S., Chiesa G. (2019). The Gut Microbiota Affects Host Pathophysiology as an Endocrine Organ: A Focus on Cardiovascular Disease. Nutrients.

[38] Sanchez-Rodriguez E., Egea-Zorrilla A., Plaza-Díaz J., Aragón-Vela J., Muñoz-Quezada S., Tercedor-Sánchez L., Abadia-Molina F. (2020). The Gut Microbiota and Its Implication in the Development of Atherosclerosis and Related Cardiovascular Diseases. Nutrients.

[39] Channappanavar R., Perlman S. (2017). Pathogenic human coronavirus infections: Causes and consequences of cytokine storm and immunopathology. Semin. Immunopathol..

[40] Tang Y., Liu J., Zhang D., Xu Z., Ji J., Wen C. (2020). Cytokine Storm in COVID-19: The Current Evidence and Treatment Strategies. Front. Immunol..

[41] Fuellen G., Liesenfeld O., Kowald A., Barrantes I., Bastian M., Simm A., Jansen L., Tietz-Latza A., Quandt D., Franceschi C. (2020). The preventive strategy for pandemics in the elderly is to collect in advance samples & data to counteract chronic inflammation (inflammaging). Ageing Res. Rev..

[42] Hojyo S., Uchida M., Tanaka K., Hasebe R., Tanaka Y., Murakami M., Hirano T. (2020). How COVID-19 induces cytokine storm with high mortality. Inflamm. Regen..

[43] Calder P.C. (2020). Nutrition, immunity and COVID-19. BMJ Nutr. Prev. Health.

[44] Lazar V., Ditu L.-M., Pircalabioru G.G., Gheorghe I., Curutiu C., Holban A.M., Picu A., Petcu L., Chifiriuc M.C. (2018). Aspects of Gut Microbiota and Immune System Interactions in Infectious Diseases, Immunopathology, and Cancer. Front. Immunol..

[45] Schirmer M., Smeekens S.P., Vlamakis H., Jaeger M., Oosting M., Franzosa E.A., Ter Horst R., Jansen T., Jacobs L., Bonder M.J. (2016). Linking the Human Gut Microbiome to Inflammatory Cytokine Production Capacity. Cell.

[46] Ferreira C., Viana S.D., Reis F. (2020). Gut Microbiota Dysbiosis-Immune Hyperresponse-Inflammation Triad in Coronavirus Disease 2019 (COVID-19): Impact of Pharmacological and Nutraceutical Approaches. Microorganisms.

[47] Kaźmierczak-Siedlecka K., Vitale E., Makarewicz W. (2020). COVID-19-gastrointestinal and gut microbiota-related aspects. Eur. Rev. Med. Pharmacol. Sci..

[48] Olaimat A.N., Aolymat I., Al-Holy M., Ayyash M., Abu Ghoush M., Al-Nabulsi A.A., Osaili T., Apostolopoulos V., Liu S.Q., Shah N.P. (2020). The potential application of probiotics and prebiotics for the prevention and treatment of COVID-19. NPJ Sci. Food.

[49] Kalantar-Zadeh K., Ward S.A., Kalantar-Zadeh K., El-Omar E.M. (2020). Considering the Effects of Microbiome and Diet on SARS-CoV-2 Infection: Nanotechnology Roles. ACS Nano.

[50] Kataoka K. (2016). The intestinal microbiota and its role in human health and disease. J. Med. Investig. JMI.

[51] Wu H.J., Wu E. (2012). The role of gut microbiota in immune homeostasis and autoimmunity. Gut Microbes.

[52] Zheng D., Liwinski T., Elinav E. (2020). Interaction between microbiota and immunity in health and disease. Cell Res..

[53] Catanzaro M., Fagiani F., Racchi M., Corsini E., Govoni S., Lanni C. (2020). Immune response in COVID-19: Addressing a pharmacological challenge by targeting pathways triggered by SARS-CoV-2. Signal Transduct. Target. Ther..

[54] Chowdhury M.A., Hossain N., Kashem M.A., Shahid M.A., Alam A. (2020). Immune response in COVID-19: A review. J. Infect. Public Health.

[55] Kalfaoglu B., Almeida-Santos J., Tye C.A., Satou Y., Ono M. (2020). T-Cell Hyperactivation and Paralysis in Severe COVID-19 Infection Revealed by Single-Cell Analysis. Front. Immunol..

[56] Zhong J., Tang J., Ye C., Dong L. (2020). The immunology of COVID-19: Is immune modulation an option for treatment?. Lancet Rheumatol..

[57] Fathi N., Rezaei N. (2020). Lymphopenia in COVID-19: Therapeutic opportunities. Cell Biol. Int..

[58] Huang I., Pranata R. (2020). Lymphopenia in severe coronavirus disease-2019 (COVID-19): Systematic review and meta-analysis. J. Intensive Care.

[59] Tan L., Wang Q., Zhang D., Ding J., Huang Q., Tang Y.-Q., Wang Q., Miao H. (2020). Lymphopenia predicts disease severity of COVID-19: A descriptive and predictive study. Signal Transduct. Target. Ther..

[60] Tavakolpour S., Rakhshandehroo T., Wei E.X., Rashidian M. (2020). Lymphopenia during the COVID-19 infection: What it shows and what can be learned. Immunol. Lett..

[61] Diao B., Wang C., Tan Y., Chen X., Liu Y., Ning L., Chen L., Li M., Liu Y., Wang G. (2020). Reduction and Functional Exhaustion of T Cells in Patients with Coronavirus Disease 2019 (COVID-19). Front. Immunol..

[62] Qin C., Zhou L., Hu Z., Zhang S., Yang S., Tao Y., Xie C., Ma K., Shang K., Wang W. (2020). Dysregulation of Immune Response in Patients with Coronavirus 2019 (COVID-19) in Wuhan, China. Clin. Infect. Dis..

[63] Huang C., Wang Y., Li X., Ren L., Zhao J., Hu Y., Zhang L., Fan G., Xu J., Gu X. (2020). Clinical features of patients infected with 2019 novel coronavirus in Wuhan, China. Lancet.

[64] Swiecki M., Colonna M. (2011). Type I interferons: Diversity of sources, production pathways and effects on immune responses. Curr. Opin. Virol..

[65] Britton G.J., Chen-Liaw A., Cossarini F., Livanos A.E., Spindler M.P., Plitt T., Eggers J., Mogno I., Gonzalez-Reiche A., Siu S. (2020). SARS-CoV-2-specific IgA and limited inflammatory cytokines are present in the stool of select patients with acute COVID-19. medRxiv.

[66] Liu Z., Long W., Tu M., Chen S., Huang Y., Wang S., Zhou W., Chen D., Zhou L., Wang M. (2020). Lymphocyte subset (CD4+, CD8+) counts reflect the severity of infection and predict the clinical outcomes in patients with COVID-19. J. Infect..

[67] Hou H., Zhang B., Huang H., Luo Y., Wu S., Tang G., Liu W., Mao L., Mao L., Wang F. (2020). Using IL-2R/lymphocytes for predicting the clinical progression of patients with COVID-19. Clin. Exp. Immunol..

[68] Dhar D., Mohanty A. (2020). Gut microbiota and Covid-19- possible link and implications. Virus Res..

[69] Geva-Zatorsky N., Sefik E., Kua L., Pasman L., Tan T.G., Ortiz-Lopez A., Yanortsang T.B., Yang L., Jupp R., Mathis D. (2017). Mining the Human Gut Microbiota for Immunomodulatory Organisms. Cell.

[70] Fontaine S.S., Kohl K.D. (2020). Optimal integration between host physiology and functions of the gut microbiome. Philos. Trans. R. Soc. B Biol. Sci..

[71] Jandhyala S.M., Talukdar R., Subramanyam C., Vuyyuru H., Sasikala M., Reddy D.N. (2015). Role of the normal gut microbiota. World J. Gastroenterol..

[72] Martin A.M., Sun E.W., Rogers G.B., Keating D.J. (2019). The Influence of the Gut Microbiome on Host Metabolism Through the Regulation of Gut Hormone Release. Front. Physiol..

[73] Budden K.F., Gellatly S.L., Wood D.L., Cooper M.A., Morrison M., Hugenholtz P., Hansbro P.M. (2017). Emerging pathogenic links between microbiota and the gut-lung axis. Nat. Rev. Microbiol..

[74] Dumas A., Bernard L., Poquet Y., Lugo-Villarino G., Neyrolles O. (2018). The role of the lung microbiota and the gut-lung axis in respiratory infectious diseases. Cell. Microbiol..

[75] Enaud R., Prevel R., Ciarlo E., Beaufils F., Wieërs G., Guery B., Delhaes L. (2020). The Gut-Lung Axis in Health and Respiratory Diseases: A Place for Inter-Organ and Inter-Kingdom Crosstalks. Front. Cell. Infect. Microbiol..

[76] Gibson P.G., Qin L., Puah S.H. (2020). COVID-19 acute respiratory distress syndrome (ARDS): Clinical features and differences from typical pre-COVID-19 ARDS. Med. J. Aust..

[77] Grasselli G., Tonetti T., Protti A., Langer T., Girardis M., Bellani G., Laffey J., Carrafiello G., Carsana L., Rizzuto C. (2020). Pathophysiology of COVID-19-associated acute respiratory distress syndrome: A multicentre prospective observational study. Lancet Respir. Med..

[78] van der Lelie D., Taghavi S. (2020). COVID-19 and the Gut Microbiome: More than a Gut Feeling. mSystems.

[79] Dickson R.P., Schultz M.J., van der Poll T., Schouten L.R., Falkowski N.R., Luth J.E., Sjoding M.W., Brown C.A., Chanderraj R., Huffnagle G.B. (2020). Lung Microbiota Predict Clinical Outcomes in Critically Ill Patients. Am. J. Respir. Crit. Care Med..

[80] Gou W., Fu Y., Yue L., Chen G.-d., Cai X., Shuai M., Xu F., Yi X., Chen H., Zhu Y.J. (2020). Gut microbiota may underlie the predisposition of healthy individuals to COVID-19. medRxiv.

[81] Zhang W., Li L., Liu J., Chen L., Zhou F., Jin T., Jiang L., Li X., Yang M., Wang H. (2020). The characteristics and predictive role of lymphocyte subsets in COVID-19 patients. Int. J. Infect. Dis..

[82] Zhao Y., Nie H.X., Hu K., Wu X.J., Zhang Y.T., Wang M.M., Wang T., Zheng Z.S., Li X.C., Zeng S.L. (2020). Abnormal immunity of non-survivors with COVID-19: Predictors for mortality. Infect. Dis. Poverty.

[83] Morrow L.E., Kollef M.H., Casale T.B. (2010). Probiotic prophylaxis of ventilator-associated pneumonia: A blinded, randomized, controlled trial. Am. J. Respir. Crit. Care Med..

[84] Zeng J., Wang C.T., Zhang F.S., Qi F., Wang S.F., Ma S., Wu T.J., Tian H., Tian Z.T., Zhang S.L. (2016). Effect of probiotics on the incidence of ventilator-associated pneumonia in critically ill patients: A randomized controlled multicenter trial. Intensive Care Med..

[85] Ceccarelli G., Scagnolari C., Pugliese F., Mastroianni C.M., d’Ettorre G. (2020). Probiotics and COVID-19. Lancet Gastroenterol. Hepatol..

[86] Mak J.W.Y., Chan F.K.L., Ng S.C. (2020). Probiotics and COVID-19: One size does not fit all. Lancet Gastroenterol. Hepatol..

